# Brain network similarity: methods and applications

**DOI:** 10.1162/netn_a_00133

**Published:** 2020-07-01

**Authors:** Ahmad Mheich, Fabrice Wendling, Mahmoud Hassan

**Affiliations:** Laboratoire Traitement du Signal et de l’Image, Institut National de la Santé et de la Recherche Médicale, Rennes, France; Laboratoire Traitement du Signal et de l’Image, Institut National de la Santé et de la Recherche Médicale, Rennes, France; Laboratoire Traitement du Signal et de l’Image, Institut National de la Santé et de la Recherche Médicale, Rennes, France

**Keywords:** Brain networks, Network similarity, Graph matching, Graph comparison

## Abstract

Graph theoretical approach has proved an effective tool to understand, characterize, and quantify the complex brain network. However, much less attention has been paid to methods that quantitatively compare two graphs, a crucial issue in the context of brain networks. Comparing brain networks is indeed mandatory in several network neuroscience applications. Here, we discuss the current state of the art, challenges, and a collection of analysis tools that have been developed in recent years to compare brain networks. We first introduce the graph similarity problem in brain network application. We then describe the methodological background of the available metrics and algorithms of comparing graphs, their strengths, and limitations. We also report results obtained in concrete applications from normal brain networks. More precisely, we show the potential use of brain network similarity to build a “network of networks” that may give new insights into the object categorization in the human brain. Additionally, we discuss future directions in terms of network similarity methods and applications.

## INTRODUCTION

The human brain is a complex network that operates at multiple time and space scales. At the macroscale, the brain can be represented as a graph where nodes denote the brain regions and edges denote the connections (structural or functional) between these regions (Bullmore & Sporns, 2009). The emerging field of network neuroscience has significantly improved our understanding about the structure and the function of the human brain (Bassett & Sporns, 2017). Graph theory, a branch of mathematics focusing on understanding systems of interacting elements, has been shown to be a very powerful tool to understand, characterize, and quantify the complex brain network (W. Huang et al., 2018; Yu et al., 2018). Applying graph theoretical measures to brain networks have revealed several nontrivial features such as small-worldness (Bassett & Bullmore, 2017), modularity (Sporns & Betzel, 2016), and scale-free (van den Heuvel, Stam, Boersma, & Pol, 2008) behaviors. The usefulness of applying graph theory and network science to brain network analysis has been widely reviewed in the last decade from methodological (da Costa, Rodrigues, Travieso, & Villas Boas, 2007; X. F. Wang & Chen, 2003) and applicative (E. Bullmore & Sporns, 2009; Christmas, Kittler, & Petrou, 1995; Cordella, Foggia, Sansone, & Vento, 2004; Hassan & Wendling, 2018; Luo & Hancock, 2001) viewpoints.

Surprisingly, much less attention has been paid to methods that quantitatively compare two graphs, a crucial issue in the context of brain networks. Comparing brain networks is indeed mandatory in several network neuroscience applications, including but not limited to (i) the estimation of similarity between structural and functional brain networks, (ii) the tracking of the temporal similarity of dynamic brain networks, and (iii) and the computation of the (dis)similarity between normal and pathological brain networks or between two conditions during a cognitive task (Avena-Koenigsberger, Misic, & Sporns, 2018; Liao, Vasilakos, & He, 2017; Paban, Modolo, Mheich, & Hassan, 2019; Rizkallah et al., 2019; Sporns, 2014).

Quantifying similarity between networks is, however, difficult because of the fact that complex networks, such as the brain, are composed of multiscale (in time and space) systems whose structure and dynamics are difficult to encapsulate in a single score. The existing graph distance measures vary depending on the features used to compute the score: nodes, edges, spatial locations, and spectrum (Christmas et al., 1995; Luo & Hancock, 2001; Mheich et al., 2018; Shimada, Hirata, Ikeguchi, & Aihara, 2016; Wilson & Zhu, 2008). Recently, several algorithms have been proposed to combine multiple features of networks when comparing two graphs (Mheich et al., 2018; Schieber et al., 2017; Shimada et al., 2016).

In this review, we will discuss the current state of the art, challenges and a collection of analysis tools that have been developed in recent years to compare brain networks. We first introduce the graph similarity problem in brain network application. We then describe the methodological background of the available metrics and algorithms of comparing graphs, their strengths and limitations. From technical viewpoint, we describe two main families of methods: (i) graph theoretical approach which consists of comparing the topological characteristics of two graphs at global, nodal, or edge-wise, mainly at group level (E. Bullmore & Sporns, 2009; Zalesky et al., 2010); and (ii) distance-based graph comparison algorithms including graphs and subgraphs isomorphism (Cordella et al., 2004), graph edit distance (Gao, Xiao, Tao, & Li, 2010), kernel approach (Shervashidze, Vishwanathan, Petri, Mehlhorn, & Borgwardt, 2009), and other approaches (Cao, Li, & Yin, 2013; Schieber et al., 2017; Shimada et al., 2016).

From an applicative viewpoint, we present new results using a recently developed algorithm called SimiNet (Mheich et al., 2018), which takes into account the physical locations of nodes when computing similarity between two brain graphs. We show the potential use of network similarity in building a “semantic map” of the brain (a network of networks).

The paper is organized as follows: we first introduce the graph similarity problem in the context of network neuroscience. Second, we introduce the graph theoretical analysis and the methods and strategies used to compute distance between networks. Third, we present new results of the application for graph similarity methods in cognitive neuroscience. Finally, we discuss certain methodological challenges and some possible future directions.

## COMPARISON BETWEEN BRAIN NETWORKS

In several domains, to understand and characterize complex systems, network construction and inference from data is crucial (Brugere, Gallagher, & Berger-Wolf, 2018; X. Liu, Kong, & Ragin, 2017; Safavi, Sripada, & Koutra, 2017). In network neuroscience, the brain graph model is an abstract mathematical representation of the interactions between brain elements (neurons, neural assemblies, or brain regions). Nodes in this graph represent neuronal assemblies or brain regions obtained from certain parcellation techniques. Edges represent either functional (statistical dependence or level of synchronization between activity patterns of brain regions) or structural links (direct anatomical connections) between neural elements (E. T. Bullmore & Bassett, 2011; Fornito, Zalesky, & Breakspear, 2013; Fornito, Zalesky, & Bullmore, 2016; Sporns, 2010). Once the brain network is built, the comparison with other brain networks can be mandatory (depending on the application).

**Figure 1.**
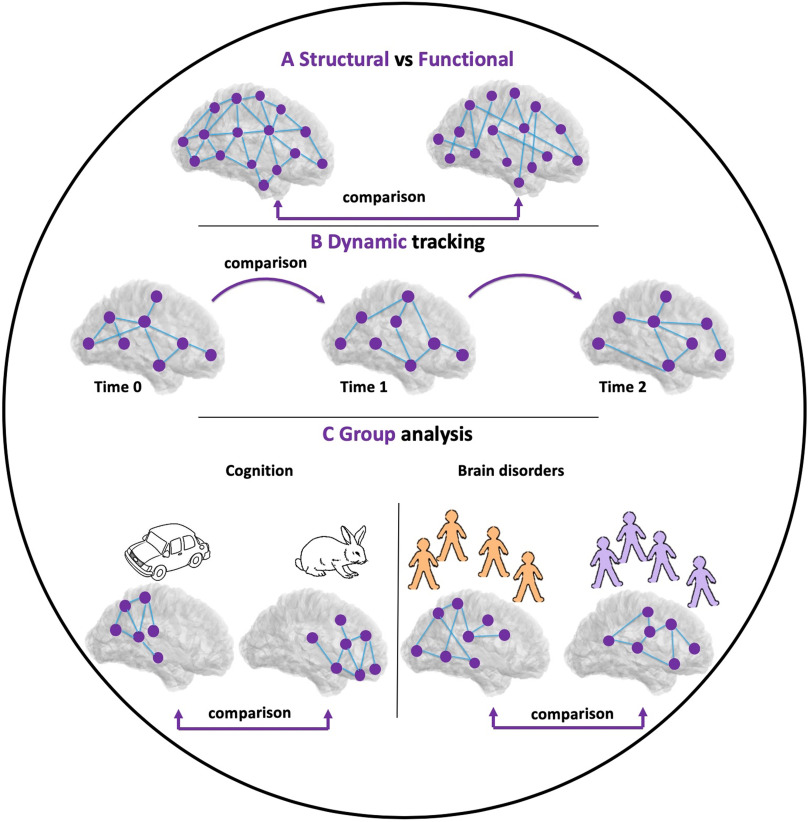
Applications of graph comparison in network neuroscience. (A) Comparison between structural and functional brain networks. (B) Tracking the dynamic of brain networks during time. (C) Comparison between two groups of brain networks for two different conditions.

Comparison techniques between brain networks have many applications because of the current widespread use of network neuroscience. These applications include, but are not limited to, (i) the statistical comparisons between brain networks for different groups of subjects, or for the same subject before and after treatment or stimulation; (ii) the discrimination between neurological disorders by quantifying functional and topological similarities (Calderone et al., 2016); (iii) the quantification of the evolution of temporal brain networks at different timescales (Hassan et al., 2015; Mheich, Hassan, Khalil, Berrou, & Wendling, 2015; O’Neill et al., 2018); (4) the comparison between real brain networks and generative network models (Figure 1); and (5) the comparison of the topological layout of nervous systems across species (van den Heuvel, Bullmore, & Sporns, 2016).

Methods and strategies used to compare brain networks can be classified into two main classes: the first one is the *statistical comparison*, where various graph theoretical metrics can be applied to characterize the topological architecture of the brain networks. The defined quantities and notations (related to graphs) used in this paper are listed in Table 1.

**Table 1.** Notation and description.

Notation	Description
*N*, *n*	Set of nodes, number of nodes
*E*, *m*	Set of edges, number of edges
*G*	Graph
*λ*	Eigenvalue
*C*	Clustering coefficient
*L*	Shortest path length
*S*	Synchronizability
*BC*	Betweenness centrality
*d*	Density
*d*_*Hamming*_(*G*′, *G*″)	Hamming distance between *G*′ and *G*″
*d*_*GED*_(*G*′, *G*″)	Graph edit distance between *G*′ and *G*″
*A*	Adjacency matrix
*Λ*	Laplacian matrix
*D*	Degree matrix
*k*_*i*_	Degree of node *i*

There are metrics of global network organization and others that can also be estimated at node or edge level of the compared networks. These metrics are then quantitatively compared between two groups of networks by using statistical tests. This class also includes spectral analysis, which witnesses an increase in its application in this field of brain networks. In the latter, the comparison is based on the eigenvalues of adjacency/Laplacian matrices of the compared graphs.

**Figure 2.**
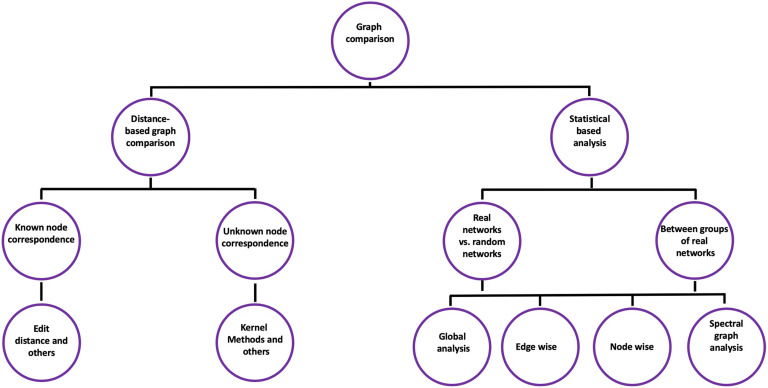
Graph comparison methods.

The second class is the distance-based graph comparison algorithms, where the main purpose is to quantify a distance (similarity score) between two networks by studying some characteristics that are considered important from an application viewpoint. Although most of the proposed algorithms are developed in specific domains, they represent promising tools to quantify similarity between brain networks (Figure 2).

## STATISTICAL COMPARISON

Statistical comparison between brain networks can be classified into two types. First is the comparison of real brain networks to random networks, where the main purpose is to validate if some characteristics of the brain networks are significantly different than chance. For a given network, a large number of random graphs can be generated, and graph theory metrics can be extracted from these random graphs, as a point of reference, to test the randomness of the same metrics measured in the real brain networks. The characteristics of these null models depend mainly on the tested hypothesis. For example, in many network neuroscience applications, null model with same degree/strength distribution as the original network are widely used (see Rubinov & Sporns, 2010). For a thorough review about null models and associated parameters, we recommend Barabási (2016) and Fornito et al. (2016).

Second, the network statistical comparison can be used to compare brain networks of two groups of subjects, such as healthy control and patients. We can classify metrics measured for comparing brain networks into four categories: global-level, node-wise, edge-wise, and spectral graph analysis (for more details about the metrics, see E. Bullmore & Sporns, 2009; Zalesky et al., 2010). In this review, we will present some of the metrics that can be used to compare two networks. However, many other metrics (or new versions of mentioned metrics) are currently available, and the reader can refer to specialized reviews about this topic. For an exhaustive review about the graph metrics, their limitations, and their applications in network neuroscience, we recommend Fornito et al. (2016). In the next section, we show a brief description of some selected metrics and their recent applications in brain network comparisons. Our purpose here is to highlight some of these metrics in respect to the main characteristics (hubness, segregation, and integration) of any given network.

### Global-Level Analysis

In this case, the graph metrics are computed over the entire network, and one value can be derived per network. Statistical tests are then applied to compare the two groups (such as healthy vs. patients).

#### Small-worldness.

Small-worldness of a network was originally introduced by Newman and Watts (1999). Other metrics associated with small-worldness, including the small-world coefficient (Humphries & Gurney, 2008), small-world metric (Telesford, Joyce, Hayasaka, Burdette, & Laurienti, 2011), small-world propensity (Muldoon, Bridgeford, & Bassett, 2016), and the small-world index (Neal, 2017), were also proposed. It is characterized by a low average shortest path length (*L*) and by a high clustering coefficient (CC). Briefly, the averaged path length *L* is defined as the average minimum number of edges that have to be traversed to pass from one node to another in the network. The CC of a node is defined as the number of existing connections between the neighbors of the node divided by all the possible connections between them. The CC quantifies the extent of local cliquishness or local efficiency of information transfer of a network.

It has been also reported that functional brain networks derived from Alzheimer’s disease patients have the characteristics of random networks with characteristic path lengths significantly longer than the healthy subjects (Stam et al., 2008). Other studies showed the presence of the small-world characteristics in the brain connectivity of healthy subjects, whereas these characteristics were disrupted in schizophrenia patients (Y. Liu et al., 2008; Lynall et al., 2010; Micheloyannis et al., 2006).

#### Modularity.

The modularity consists of partitioning a network into a number of nonoverlapping groups or modules, also called communities (Rubinov & Sporns, 2010). Network modules are defined by a subset of nodes in the graph that are densely intraconnected and weakly connected to other nodes (Girvan & Newman, 2002; Sporns & Betzel, 2016). Several methods have been proposed to resolve the community structure of complex networks in several applications (Blondel, Guillaume, Lambiotte, & Lefebvre, 2008; Lancichinetti & Fortunato, 2009; Newman, 2006). For brain networks applications, the modularity maximization method (Newman & Girvan, 2004) is the most applied in the detection of brain networks modules. The main idea of this method is to split the nodes of a network into K nonoverlapping communities in order to maximize the modularity quality function *Q*. A minimum value of *Q* near to 0 indicates that the network considered is close to a random one, whereas a maximum value of *Q* near to 1 indicates a strong community structure.

A number of studies have found significant differences between the brain networks of two groups by comparing their modules. Meunier, Lambiotte, and Bullmore (2010) investigated the modular structure of the human brain networks derived from fMRI measurements for two groups of younger and older adults by using a modularity maximization algorithm. The results showed that the brain becomes less modular with age, a finding also reported by others (Baum et al., 2017). In brain diseases applications, Alexander-Bloch et al. (2010) showed that modularity decreases for schizophrenia patients compared with healthy subjects. In turn, Peraza, Taylor, and Kaiser (2015) revealed an increase in modularity for patients with Lewy body disease. Other techniques like the allegiance matrix (Mattar, Cole, Thompson-Schill, & Bassett, 2015) and scaled inclusivity (Moussa, Steen, Laurienti, & Hayasaka, 2012; Steen, Hayasaka, Joyce, & Laurienti, 2011) were also proposed to explore the consistency of community structure in brain networks.

#### Efficiency.

The network efficiency quantifies the exchange of information across the whole network. It is defined as the inverse of the average path length (Achard & Bullmore, 2007; Latora & Marchiori, 2001). Global efficiency was used to compare functional brain networks between two groups of healthy old and healthy young subjects (Achard & Bullmore, 2007) where authors showed a reduction in the efficiency for older people. In addition, several studies showed that patients with schizophrenia, Alzheimer’s and Parkinson’s diseases had a noticeable reduction in global efficiency compared with healthy controls (Berlot, Metzler-Baddeley, Ikram, Jones, & O’Sullivan, 2016; Reijmer et al., 2013; Skidmore et al., 2011).

### Node-Wise Analysis

In this case, the graph metrics are calculated for each node, and then the node’s metric values are compared between the two graphs. The main advantages of such an approach are (i) the possibility to explore more features in the graph, (ii) the presence of more data (number of nodes) to compare between conditions, and (iii) this comparison will not only show if there is a difference between two conditions but will also indicate where the difference is located (on which brain regions). However, it can produce false positive results as the activities of the nodes are not fully independent. This type of analysis required correction for multiple comparisons, as comparison was done n (number of nodes) times, using methods such as Bonferroni (Rice, 1989) or false discovery rate (FDR) (Genovese, Lazar, & Nichols, 2002). Several metrics can be computed at the level of network’s nodes. For detailed and comprehensive review, we recommend Rubinov and Sporns (2010). Globally speaking, these metrics reflect mainly three behaviors in the network: segregation, integration, and hubness.

#### Segregation.

Segregation is the ability of the network to do specialized processing in a densely interconnected group of nodes. This includes measures such as (i) clustering coefficient, which is defined by the fraction of the nodes’ neighbors that are also neighbors of each other (Watts & Strogatz, 1998); (ii) local efficiency, which is a measure of the efficiency of information transfer limited to neighboring nodes—it is calculated as the average nodal efficiency among the neighboring nodes of node *i*, excluding node *i* itself; and (iii) intramodule degree, which denotes how well connected is a node compared with other nodes of the same community. Chan et al. found a decrease in segregation of brain network when age increases (Chan, Park, Savalia, Petersen, & Wig, 2014). The network segregation was shown to be improved in Alzheimer’s and schizophrenia patients (He, Chen, & Evans, 2008; Kabbara et al., 2018; Yao et al., 2010; Y. Zhang et al., 2012) and reduced for epilepsy patients (Z. Zhang et al., 2011).

#### Integration.

Integration is the ability of the network to combine information from distant nodes. This includes measures such things as (i) participation coefficient, which quantifies the balance between the intramodule versus intermodule connectivity for a given node, and (ii) characteristic path length, which is defined as the average shortest path length between all pairs of nodes in a network (Watts & Strogatz, 1998). Several studies were performed to compare brain networks of healthy subjects and Alzheimer’s patients (Stam, Jones, Nolte, Breakspear, & Scheltens, 2006; Supekar, Menon, Rubin, Musen, & Greicius, 2008), and results showed an increase of characteristic path length in patients.

#### Hubness.

This include measures such qualities as (i) strength, which describes the connection strength of node to all other nodes, and (ii) betweenness centrality, defined as the fraction of all shortest paths in the network that pass through a given node. Many studies showed that brain disorders, such as Alzheimer’s disease, coma, and schizophrenia, are associated with alterations in hubs, (Achard et al., 2012; Bassett et al., 2008; Crossley et al., 2014; He et al., 2008; Lynall et al., 2010; van den Heuvel, Mandl, Stam, Kahn, & Pol, 2010). C. Yan et al. (2010) used betweenness centrality to investigate the effects of sex on the topological organization of human cortical anatomical network. In clinical application, betweenness centrality was used to compare brain networks of healthy subjects and patients with schizophrenia, depression, and Alzheimer’s disease (Becerril, Repovs, & Barch, 2011; van den Heuvel et al., 2010; Yao et al., 2010).

Other studies have compared brain networks of healthy subjects and patients diagnosed with schizophrenia by using the degree of nodes (Bassett et al., 2008; Lynall et al., 2010). The results showed a reduced degree in several brain nodes of schizophrenia patients. Other studies showed also that Parkinson’s disease patients have a significant decrease in the degree of several brain regions in their functional network, such as left dorsal lateral prefrontal cortex (Wu et al., 2009).

### Edge-Wise Analysis

Edge-wise analysis consists of calculating a statistical test (such as Student’s t test) on each edge in the graph. If the number of nodes in a graph are equal to n, then the maximum number of edges (in the case of undirected network) is equal to (*n* × (*n* − 1)/2). The statistical test is calculated (*n* × (*n* − 1)/2) times. This method also requires correction for multiple comparisons by using methods such as Bonferroni or FDR. Other approaches have also been proposed to deal with the family-wise error rate, such as the network-based statistic (NBS) method (Zalesky et al., 2010). The main idea of this method (based on permutation analysis) is to find a network “pattern” (a set of nodes connected by edges) instead of a single link that differentiates the two conditions. NBS has been widely used to identify alterations in brain networks associated with psychiatric disorders such as schizophrenia and depression (Zalesky et al., 2011), and to identify cognitive phenotypes in Parkinson’s disease patients (Hassan et al., 2017).

### Spectral Graph Analysis

**Figure 3.**
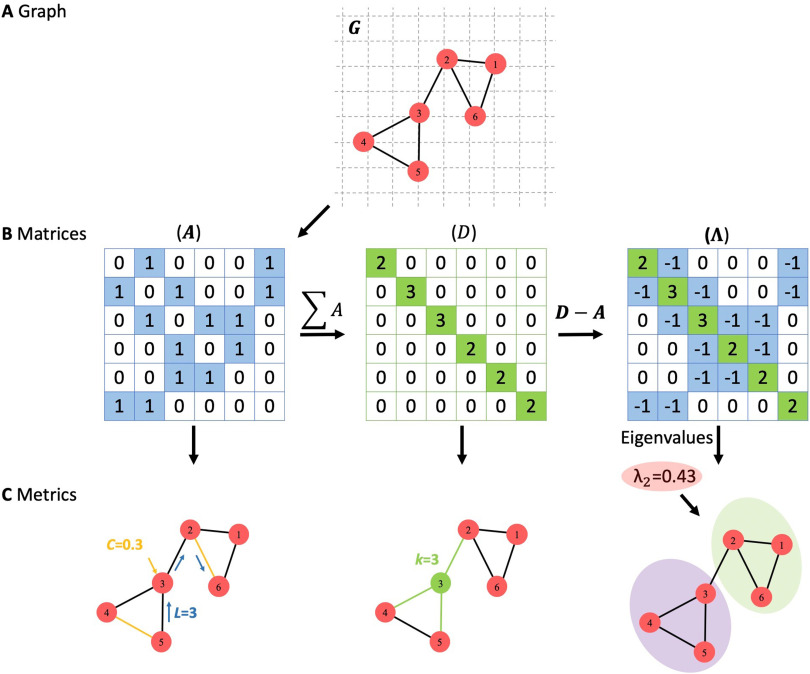
(A) A graph with six nodes and seven edges. (B) Adjacency matrix (A), degree matrix (D), and Laplacian matrix (∧). (C) Some of graph metrics extracted from each matrix, where *C* represents the clustering coefficient of node 3, *L* is the shortest path length between node 5 and node 6, *k* represents the degree of node 3, and *λ*_2_ is the second eigenvalue of the graph *G*.

Spectral graph theory is a branch of graph theory which has been widely used to characterize the properties of a graph and extract information about its structure. For a graph *G*(*N*, *E*) of *n* nodes with adjacency matrix *A*_*n*×*n*_ and degree matrix *D*_*n*×*n*_, the Laplacian matrix Λ_*n*×*n*_ is computed using the following formula (Figure 3):

$$\Lambda (i,j)=\left\{\begin{array}{c} D_{i}\;for\;i=j\\ -A(i,j)\;for\;i\neq j \end{array}\right.\;where\;i,j\in N$$

Once the Laplacian matrix is constructed, the eigenvalue of *G* can be computed (*λ*_1_, *λ*_2_ … *λ*_*n*_). Spectral graph analysis is well known in many domains for its powerful characterization of network properties (Banerjee, 2012; Farkas et al., 2002). It provides important information on relevant network properties, such as connectivity levels, resilience to damage, and the spread of information throughout the network (de Haan et al., 2012). Comparing brain networks by using spectral graph theory was recently performed in several studies, including the comparison of network’s eigenvalue distributions over the structural brain networks of different species, such as caenorhabditis elegans, macaque, and cat (de Lange, de Reus, & van Den Heuvel, 2014). It was also used to detect network alterations in patients with Alzheimer’s disease (de Haan et al., 2012).

#### Synchronizability.

Synchronizability (*S*) quantifies the robustness of the network with respect to edge removals. It is computed as the report between the second smallest eigenvalue and the highest eigenvalue of the Laplacian matrix of the network.

$$S=\frac{\lambda_{2}}{\lambda_{n}}$$

A network with low value of *S* is more vulnerable to disconnection. In return, a high *S* value means less vulnerable to disconnection. Several studies showed that many graph properties such as clustering coefficient, average distance, average degree, and degree distribution failed to characterize the synchronizability of networks. In return, the spectral analysis can detect this synchronizability for the same network (Atay, Bıyıkoğlu, & Jost, 2006).

**Figure 4.**
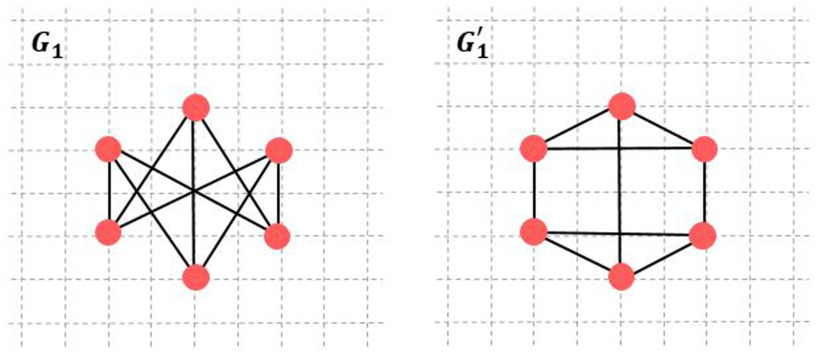
Graph *G* and *G*′.

For example, two graphs *G*_1_ and *G*_1_′ have the same number of nodes (*n* = 6) and edges (*m* = 9), shown in Figure 4. These two graphs share common statistical network metrics such as: density, betweenness centrality, average degree, and global efficiency (Table 2), but they are different in their S: *λ*_2_(*G*_1_) = 3 and *λ*_2_(*G*_1_′) = 2, then *S*(*G*_1_) = 3/6 and *S*(*G*_1_′) = 2/5.

**Table 2.** Depiction of graph metrics.

Graphs	Density	BC	Degree	Global Efficiency	*S*
*G*_1_	0.6	2	3	0.8	0.5
*G*_1_′	0.6	2	3	0.8	0.4

*Note*. BC = betweenness centrality; S = synchronizability.

De Haan et al. (de Haan et al., 2012) used graph spectral analysis to study synchronizability between healthy subjects and patients with Alzheimer’s disease. Results showed a decrease in synchronizability and loss of network connectivity in all frequency bands for Alzheimer disease patients.

## DISTANCE-BASED GRAPH COMPARISON

The main idea of distance-based graph comparison methods consists of comparing two graphs and providing a “similarity” score. This similarity value (if normalized) ranges from 0 (no similarity at all) to 1 (fully similar/same network). Distance-based graph comparison includes two families of methods:

1.  *Known node correspondence*. This includes methods based on *edit distances* that focus on common and uncommon elements (nodes and edges), such as graph edit distance (GED) and hamming distance (Gao et al., 2010). They also include more elaborate techniques such as DeltaCon (Koutra, Vogelstein, & Faloutsos, 2013) and SimiNet (Mheich et al., 2018).
2.  *Unknown node correspondence*. This includes, for instance, *kernel methods* (Borgwardt & Kriegel, 2005; Shervashidze, Schweitzer, Leeuwen, Mehlhorn, & Borgwardt, 2011) that focus on the *structure* of the networks by comparing their Laplacian matrices and methods that use nodeinvariant graph statistics to compare graphs (Figure 2).

### Algorithms Based on Edit Distance

Quantifying the similarity/distance between two brain networks by using edit distance algorithms allows one to find the common/uncommon nodes (brain regions) and edges (functional/structural) between two brain networks.

#### The hamming distance.

The hamming distance is the most direct way to compare two networks (Deza & Deza, 2013). It is defined as the sum of difference between the adjacency matrices of two networks *G*′ and *G*″:

$$d_{Hamming}\left(G^{\prime },G^{\prime \prime }\right)=\underset{i\neq j}{\sum }A_{ij}^{\prime }\neq A_{ij}^{\prime \prime }$$

where *i* and *j* are two nodes, and *A*′ and *A*″ are the adjacency matrices of *G*′ and *G*″, respectively.

#### Graph edit distance.

GED is another popular distance between two networks (Gao et al., 2010), widely applied in several applications (X. Wang, Ding, Tung, Ying, & Jin, 2012; Zeng, Tung, Wang, Feng, & Zhou, 2009). It is defined as the minimum-weight sequence of edit operations required to transform one graph into another (edit operations on a graph are insertion, deletion, or substitution applied on both nodes and edges). The GED between two graphs *G*′ and *G*″ is defined as:

$$d_{GED}\left(G^{\prime },G^{\prime \prime }\right)=min\underset{u\in U}{\sum }c(e_{u})$$

where *c*(*e*_*u*_) is the cost of an edit operation from *G*′ to *G*″, and *U* is the total number of edit operations. A difficult step in this approach is to define the cost function for different operations.

#### SimiNet algorithm.

**Figure 5.**
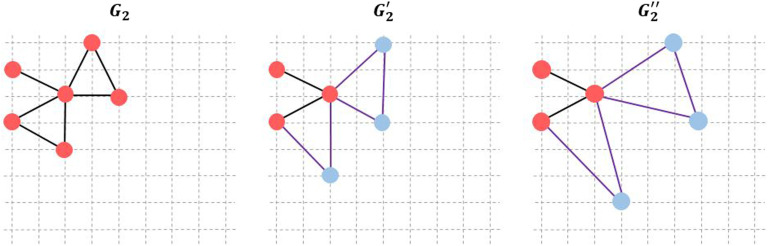
Three networks with the same number of nodes (6) and edges (7) located into a grid (8 × 8).

An important characteristic, not integrated in the previous approaches, is the spatial location of nodes, which denotes the 3D coordinates of the brain regions. The physical location of nodes can add additional key information when measuring similarity between brain networks. For instance, two networks with identical properties but interconnecting distant brain regions can have low similarity. Conversely, two networks with dissimilar properties but interconnecting spatially close brain regions can be very similar. In this context, SimiNet explores both the nodes and the edges when computing the similarity index. Concerning the nodes, the algorithm is based on four main steps: (i) detection of common nodes between the two compared graphs, (ii) substitution between two nodes where the cost of substitution is equal to the distance between the substituted nodes, (iii) insertion for new nodes where the cost of insertion is equal to a constant value, and (iv) deletion of nodes where the cost of suppression is equal to the cost of insertion. The (cost (substitution) < cost (insertion) + cost (deletion)) is always preserved. The second step is to calculate the edges distance. It consists of calculating the sum of the weight difference between two edges of two compared graphs. The algorithm provides a normalized similarity index (SI): 0 for no similarity and 1 for two identical networks (same properties and topology). The algorithm is detailed and compared with other methodologies in Mheich et al. (2018). Figure 5 displays three graphs, *G*_2_, *G*_2_′, and *G*_2_″, with the same number of nodes (*n* = 6) and edges (*m* = 7); these graphs are located on a grid (8 × 8). Graphs *G*_2_′ and *G*_2_″ are obtained by shifting three nodes of *G*_2_ randomly. The similarity score is then calculated between the three graphs by using the SimiNet, hamming, and GED algorithms (Table 3). As can be seen in this example, Hamming and GED do not capture the spatial shifting of nodes, which is the case with SimiNet.

**Table 3.** Depiction of similarity scores between the three networks (*G*_2_, *G*_2_′, and *G*_2_″) by using SimiNet, Hamming distance, and graph edit distance (GED) algorithms.

Graphs	SimiNet	Hamming	GED
(*G*_2_, *G*_2_′)	0.8	0.38	0.55
(*G*_2_, *G*_2_″)	0.6	0.38	0.55
(*G*_2_′, *G*_2_″)	0.7	0.38	0.55

### Algorithms Based on Structure Distance

Computing the similarity/distance between two brain networks by using algorithms that prioritize network structures allow us to spot and to quantify structural topology differences such as the presence or absence of important edges, nodes, cliques, or subgraphs that have influence on the information flow through the network. Several algorithms have been proposed to compute the network similarity based on structure distance. Some of these approaches and algorithms are briefly described hereafter.

#### DeltaCon algorithm.

The DeltaCon algorithm assesses the similarity for same-size networks (two networks with same number of nodes) (Koutra et al., 2013). The idea of this method is to compute the matrix of pairwise node affinities in the first network and to compare them with the one in the second network, where node affinities is the influence of each node on the other network’s nodes. The difference between the matrices is then computed to produce an affinity score measuring the similarity between the compared networks. Readers may refer to Koutra et al. (2013) for more details about the DeltaCon algorithm steps and implementation. This algorithm also provides a normalized similarity value ranging from 0 (dissimilar graphs) to 1 (identical graphs). DeltaCon satisfies some important network properties: (i) Edge importance, where edges that connect two components are of higher cost than other edges; (ii) Weight awareness for weighted networks, where changes on edges with high weight values have more impact on the final similarity score; and (iii) Edge submodularity, where a specific change on a network with few edges is more important than that in a denser network.

#### D-measure.

Recently, Schieber et al. (2017) proposed a new algorithm to quantify graph dissimilarities. The dissimilarity score is bordered between 0 and 1, where a larger score corresponds to more dissimilar graphs, and a lower score to more similar graphs. The score produced by the algorithm is based on a combination of three components: (i) dissimilarity in average node connectivity; (ii) dissimilarity in a node dispersion metric, where node dispersion for a graph measures the distribution of distances between nodes in this graph and allows to make comparison with node dispersion in the second graph; and (iii) dissimilarity in node *α*-centrality (measure of nodes centrality within a graph). The main advantage of this algorithm is the ability to detect structural differences such as critical edges (connect two components) that have an influence on the information through the graph. D-measure was applied to brain networks in order to compare two groups of subjects (39 control and 68 alcoholic samples) (Schieber et al., 2017). The algorithm was able to find the brain networks that discriminate control and alcoholic participants.

#### Kernel methods.

Graph kernel methods are based on first mapping the graphs into a higher dimensional feature space, and then searching for the common features among the mapping graphs. Given two graphs *G*_3_ and *G*_3_′, the basic idea behind graph kernel is to construct a kernel *ξ*(*G*_3_, *G*_3_′) = 〈*ϕ*(*G*_3_), *ϕ*(*G*_3_′)〉 where the similarity score between *G*_3_ and *G*_3_′ value corresponds to the scalar product between the two vectors *ϕ*(*G*_3_) and *ϕ*(*G*_3_′) in a Hilbert space. Several graph kernels–based algorithms have been proposed to measure networks similarity such as random walks, shortest paths, and Weisfeiler–Lehman.

A random walk kernel counts the number of matching labeled random walks (Vishwanathan, Schraudolph, Kondor, & Borgwardt, 2010). The matching between two nodes is determined by comparing their attribute values. The measure of similarity between two random walks is then defined as the product of the kernel values corresponding to the nodes encountered along the walk.

Shortest path kernel computes the shortest path kernel for a set of graphs by exact matching of shortest path lengths (Borgwardt et al., 2005). The Floyd–Warshall algorithm (Floyd, 1962) is usually used to calculate all the pairs shortest paths in *G*_3_ and *G*_3_′. The shortest path kernel is then defined by comparing all the pairs of the shortest path lengths among nodes in *G*_3_ and *G*_3_′.

**Figure 6.**
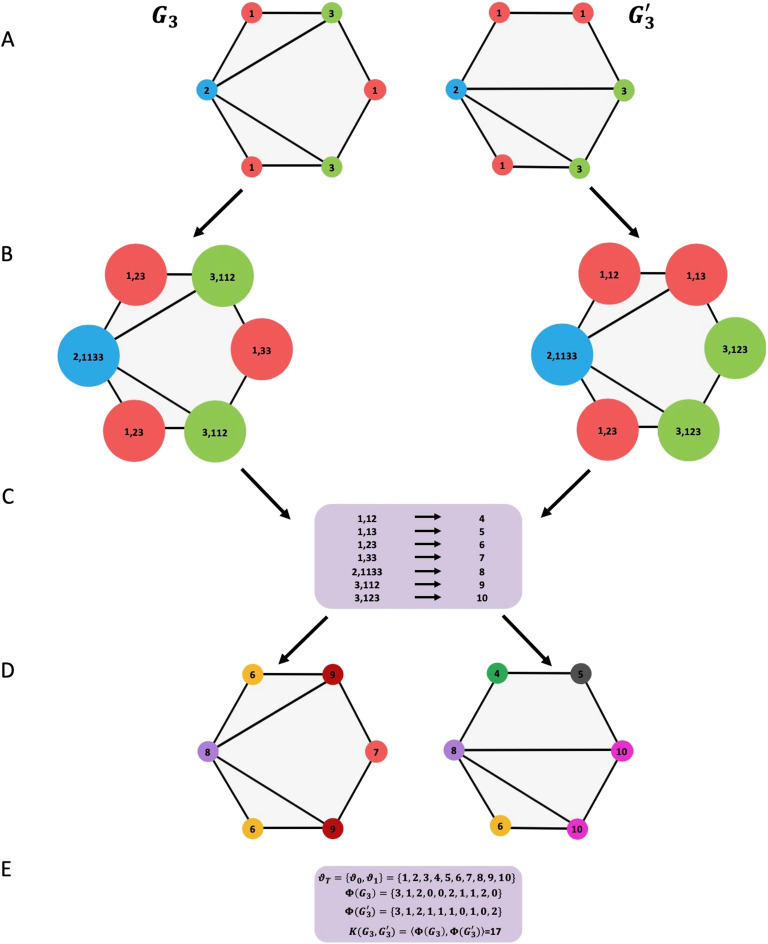
Illustration of the construction process of the Weisfeiler–Lehman subtree kernel with *h* = 1 for two graphs *G*_3_ and *G*_3_′. (A) The initial labeled graph *G*_3_ and *G*_3_′. (B) Augmented label on graph *G*_3_ and *G*_3_′. (C) Label compression. (D) Relabeled graph. (E) Computation of the kernel on graph *G*_3_ and *G*_3_′, where *ϑ*_0_ is the set of original node labels and *ϑ*_1_ the set of compressed node labels; *ϕ*(*G*_3_) and *ϕ*(*G*_3_′) are the count of node labels.

Weisfeiler–Lehman (Shervashidze et al., 2011) computes h-step Weisfeiler–Lehman kernel for a set of graphs. The main idea of this algorithm is to increase the node labels by the sorted set of node labels of neighboring nodes and compress these increased labels into new shorted labels. These steps are repeated until the node label sets of *G*_3_ and *G*_3_′ differ or the number of iterations reaches a maximum *h*. A detailed example is presented in Figure 6.

### Other Graph Comparison Approaches

Some other approaches are indirectly related to graph similarity and may help to tackle some of the graph similarity challenges. One of these approaches is “graph classification” in which the main idea is to classify individual graphs into two or more categories based on graph features comparison (Heimann, Safavi, & Koutra, 2019). A number of deep-learning algorithms have been introduced also to classify graphs in different fields, such as artificial intelligence, image analysis, and neuroscience (S. Wang et al., 2017; Y. Yan et al., 2019; M. Zhang, Cui, Neumann, & Chen, 2018). Recently, based on latent graph feature/embedding comparison, Heimann et al. (2019) proposed a randomized grid mapping that captures the distribution of a graph’s node embeddings at multiple levels of resolution. The difference between similarity approaches and these classification methods is that the latter does not necessarily produce a similarity score as an output, but they can directly separate networks into classes, and thus can be very useful for some neuroscience applications. In network neuroscience, several machine/deep-learning approaches were developed to learn latent features or extract meaningful information embedded in networks (Hinton & Salakhutdinov, 2006; A. Huang, 2008; Kamiya et al., 2015; Oh Song, Xiang, Jegelka, & Savarese, 2016). For instance, Kawahara et al. (2017) proposed a new framework called BrainNetCNN that allows one to make predictions from brain networks such as the prediction of brain network development (H. Li, Satterthwaite, & Fan, 2018; X. Li, Li, & Li, 2017.

## NETWORK OF NETWORKS

**Figure 7.**
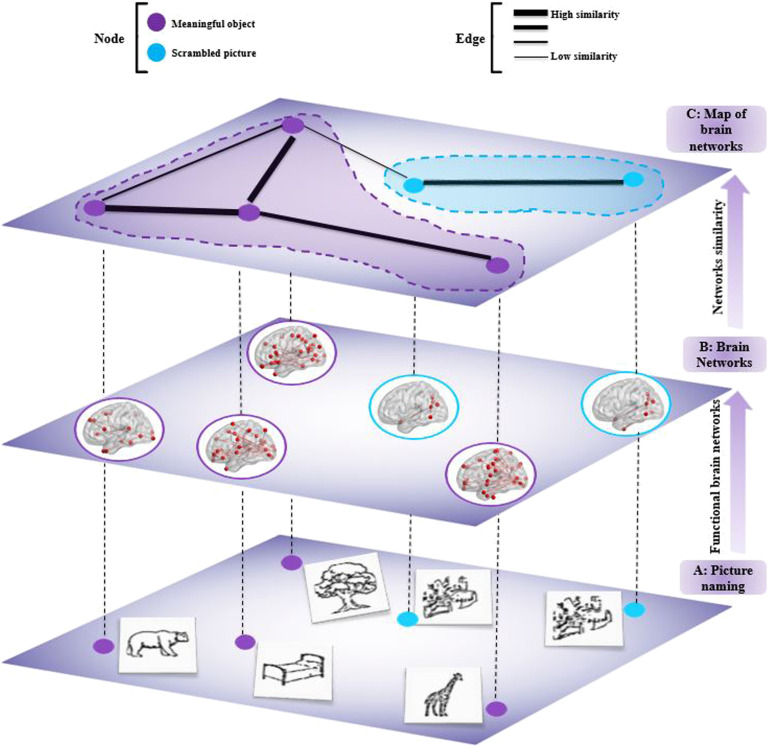
A schematic representation of the proposed method. (A) Signal recording during meaningful and scrambled pictures naming task. (B) Estimating the functional brain network for each picture. (C) Measurement of the similarity between brain networks using SimiNet and classify them into meaningful and scrambled pictures.

Analyzing similarity between brain networks can be useful for several applications in cognitive and clinical neuroscience. Here, we show an example of its application to functional networks estimated during visual object recognition task. To do so, we used dense electroencephalography (256 electrodes) data from 20 subjects who were asked to name two categories of pictures (39 meaningful and 39 scrambled). Then, we construct a map based on the similarity scores between brain functional networks (Figure 7). This data is described in Mheich et al. (2018) and approved by the National Ethics Committee for the Protection of Persons, Braingraph study, agreement number (2014-A01461-46), and promoter, Rennes University Hospital.

**Figure 8.**
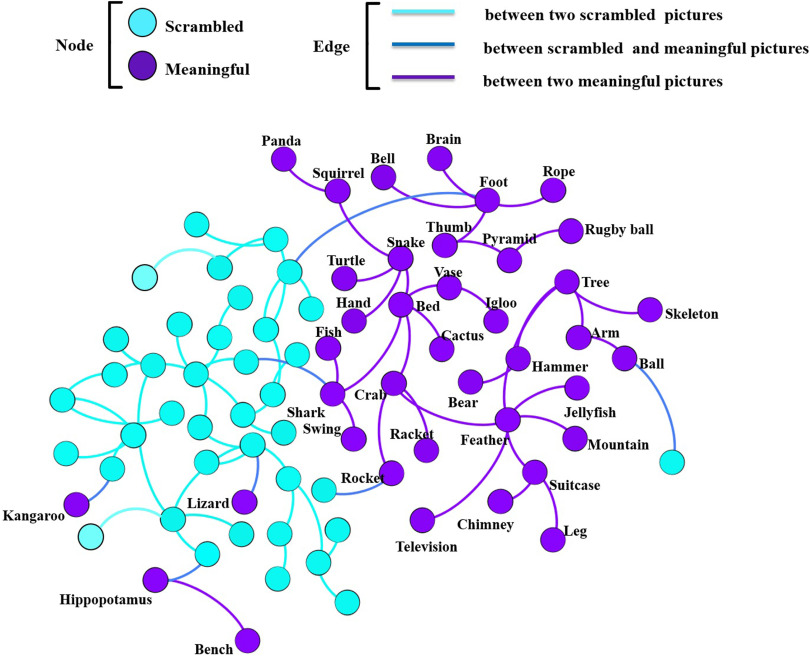
Network of brain networks. The purple nodes represent meaningful objects, and the blue nodes represent scrambled objects. The purple edges represent a high similarity value between two functional brain networks of meaningful category. The blue edges represent a high similarity value between two functional brain networks of scrambled category, and the dark blue edges represent a high similarity value between two brain networks of meaningful and scrambled objects.

Functional brain network for each object (picture) was constructed at the cortical level by using the EEG–source connectivity method (Hassan & Wendling, 2018). The similarity scores between all the object-related functional networks were quantified using the SimiN et algorithm, which produce a 78 × 78 similarity matrix. The similarity matrix was transformed into a graph where nodes represent brain networks and edges represent the highest similarity score between the brain networks. This graph is illustrated in Figure 8. The visual inspection of this graph (blue nodes for meaningful and purple nodes for scrambled) shows that the connections between objects of the same category (*N* = 72) is clearly higher than connections between objects from different categories (*N* = 7). Constructing this *network of networks* can be seen as a first attempt to evaluate categorization of visual objects in the human brain with a functional network similarity-based approach.

## DISCUSSION AND CONCLUSIONS

As long as there are functional/structural brain networks, there will be people looking for comparisons between them. Here, we have presented the main methods and algorithms that can be used to compare brain networks.

Which method then? The answer depends on the application itself. If the objective is to reveal statistical difference between two groups (healthy subjects vs. patients, for instance) then the methods based on the graph theoretical approach (node-wide or edge-wise) can be good candidates provided that physiological hypothesis are well set and statistical parameters are carefully chosen (correction for multiple comparisons, for instance).

However, if the objective is to produce a similarity score (usually normalized between 0 and 1), then the distance-based graph comparison methods are more appropriate. Validation of algorithms, like the comparative analysis of Mheich et al. (2018), can allow us to identify a set of methods that perform well on simulated networks. However, we do not know how well real networks are described by currently used simulations.

Therefore, there is no guarantee that methods performing well on benchmarks also give reliable results on real brain networks (advantages and limitations of some of these algorithms are presented in Table 4).

**Table 4.** Advantages and limitations of some selected graphs distance measures.

		Characteristics				
		Spatial location	Different size	Computational cost	Structural difference	Available Code
Methods	SimiNet (Mheich et al., 2018)	+	−	++	−	https://github.com/amheich/SimiNet
	D-measure (Schieber et al., 2017)	−	−	+ (not for sparse graph)	+	https://github.com/tischieber/Quantifying-Network-Structural-Dissimilarities
	DeltaCon (Koutra et al., 2013)	−	−	+	+	https://web.eecs.umich.edu/∼dkoutra/CODE/deltacon.zip
	Kernel methods (Borgwardt et al., 2005; Shervashidze et al., 2011)	−	+	− −	+	https://github.com/BorgwardtLab/GraphKernels

*Note*. Note that “−” indicates a characteristic that is not integrated in the similarity score of the method; “+” a characteristic that is integrated in the methods; “− −” for worst computational time; and “++” for very good computational time. Spatial location = physical location of nodes; Different size = graphs with different number of nodes; Computational cost = algorithm running time; Structural difference = detection of difference between node’s links in two graphs.

From an application viewpoint, the network similarity is crucial in the identification of what we called here “network of networks.” This can be used to build a “semantic map” where nodes can represent the estimated networks of visual/auditory objects and edges can denote the similarity between these networks (preliminary results are presented in this review). This will undoubtedly require a very large number of stimuli and also the repetition of each stimuli several times. When these conditions are respected, these semantic maps can give new insights into the object categorization process in the human brain, from a network-based perspective.

In clinical neuroscience, a potential application of network distance measures is the mapping of a “disease network” where the nodes may represent each brain disease and the edges can represent the similarity between the different networks associated to each disease (such as Parkinson’s, Alzheimer’s, epilepsy, and so on). This application could help to further understand the possible common altered network patterns in brain disease. A very recent review by van den Heuvel and Sporns (2019a) showed indeed the importance of investigating such cross-disorder connectivity patterns.

Another potential application of the network of networks approach is to construct a similarity network across species connectomes (van den Heuvel et al., 2016), in which nodes can denote species and edges the similarity between them. The major difficulty of this application is to have access to connectome data from a range of species (human, drozophila, *C. elegans*, cat, macaque, pigeon, mouse, rat, etc.). This may help to better understand cross-species communalities and differences in term of brain structure and function. Moreover, a combination of hypothesis-based selection of graph metrics (specific hubs, modules, etc.) with the similarity algorithms may also improve the categorization and the classifications of these subnetworks.

### Some Challenges in Brain Network Similarity

First, in the statistical comparison (graph theoretical–based approach), the major difficulty arises from the fact that graph measures depend on the number of nodes and edges. To compare two different brain networks, choosing equal size and density has become more popular so that differences in graph measures appear solely through structural changes (van Wijk, Stam, & Daffertshofer, 2010). However, this can be only achieved by taking a fixed number of nodes and imposing a desired average degree by adjusting the binary threshold (van Wijk et al., 2010).

Second, knowing the graph metrics that enable one to detect the difference between brain networks is not obvious. The choice of this graph metric is often empirical. For a more appropriate approach, these graph metrics should be driven by the physiopathology of the analyzed neuroscience question or by adopting methods based on Network Representation Learning (NRL). Indeed, these NRL approaches avoid the necessity for thorough feature engineering and have led to very important results in network-based tasks, such as node classification, node clustering, and prediction (D. Zhang, Yin, Zhu, & Zhang, 2018). We believe that NRL could be very useful to the network neuroscience community for the adaption of representation learning techniques to specific applications that are of interest in the field. In addition, a key challenge is to encapsulate several graph metrics in one similarity score that can describe the difference between two graphs without missing any characteristics.

Third, the distance-based graph algorithms developed in different fields are indeed very useful for detecting uncaptured characteristics by graph metrics. However, these algorithms are still limited to undirected graphs. More efforts are needed to expand these algorithms to deal with directed (causal) brain networks.

### Possible Future Directions

From a methodological viewpoint, more efforts are needed to develop and optimize similarity algorithms that combine several graph characteristics into one similarity score. These algorithms should be first analyzed/validated using simulated data (where ground truth can be, to some extent, obtained) before applying them to real brain networks. Another methodological approach is to use similarity methods in brain dynamic algorithms in order to decipher how brain networks change over time. In most dynamic analysis algorithms, a similarity/correlation step is always needed to compare adjacent networks. This is usually done by using the classical correlation coefficient (O’Neill et al., 2018). Adding a network-based similarity index into brain dynamic algorithms can dramatically improve their performance.

From an application viewpoint, an interesting future clinical application is the construction of a “network of brain diseases,” where nodes can represent brain diseases and edges represent the similarity score between them. This map may help to characterize and visualize the common landscapes between brain disorders, an issue recently reviewed by van den Heuvel and Sporns (2019b).

We hope that the survey will motivate more researchers to contribute with other ideas than those described above in the brain network similarity field, from a methodological and/or an applicative perspective.

## AUTHOR CONTRIBUTIONS

Ahmad Mheich: Conceptualization; Writing - Original Draft; Writing - Review & Editing. Fabrice Wendling: Supervision. Mahmoud Hassan: Conceptualization; Supervision; Writing - Review & Editing.

## TECHNICAL TERMS

Graph: A mathematical description of a network comprising a set of nodes and a set of edges representing the pairwise relations between nodes.
Graph theory: A branch of mathematics that studies the structural organization of graphs.
Functional brain networks: A statistical relation between time series of physiological activity of neural assemblies (e.g., brain regions).
Distance-based graph comparison algorithms: Methods that produce a distance or a similarity score in order to compare graphs.
Graphs isomorphism: Graphs that have the same number of nodes and that are connected in the same way.
Graph edit distance: A graph similarity method defined as the minimum cost of edit operations to transform one graph to another.
Spectral analysis: A branch of graph theory that studies the spectrum of eigenvalues and eigenvectors of the adjacency matrix of the network.
Adjacency matrix: A matrix that describes the absence or presence of a connection between all pairs of nodes in a graph.
Laplacian matrix: A matrix that carries important properties about a graph, many relating to its spectrum.
Graph kernel methods: A set of functions measuring the similarity between graphs based on the inner product.
